# Old but gold: the role of drug combinations in improving response to immune check-point inhibitors in thoracic malignancies beyond NSCLC

**DOI:** 10.37349/etat.2021.00030

**Published:** 2021-02-28

**Authors:** Luca Cantini, Federica Pecci, Filippo Merloni, Andrea Lanese, Edoardo Lenci, Francesco Paoloni, Joachim G.J.V. Aerts, Rossana Berardi

**Affiliations:** 1 Clinical Oncology, Università Politecnica delle Marche, A.O.U. Ospedali Riuniti, 60126 Ancona, Italy; 2 Department of Pulmonary Medicine, Erasmus MC, 3015 CE Rotterdam, The Netherlands; 3 Erasmus MC Cancer Institute, Erasmus MC, 3015 CE Rotterdam, The Netherlands; Istituto Nazionale Tumori-IRCCS-Fondazione G. Pascale, Italy

**Keywords:** Immune checkpoint inhibitors, small cell lung cancer, mesothelioma, thymic epithelial tumor, immuno-oncology, immune modulating

## Abstract

The introduction of immune checkpoint inhibitors (ICIs) in non-oncogene addicted non-small cell lung cancer (NSCLC) has revolutionized the treatment scenario and led to a meaningful improvement in patient prognosis. Disappointingly, the success of ICI therapy in NSCLC has not been fully replicated in other thoracic malignancies as small cell lung cancer (SCLC), malignant pleural mesothelioma (MPM), and thymic epithelial tumors (TETs), due to the peculiar biological features of these disease and to the difficulties in the conduction of well-designed, biomarker-driven clinical trials. Therefore, combination strategies of ICIs plus conventional therapies (either chemotherapy, alternative ICIs or targeted agents) have been implemented. Although first approvals of ICI therapy have been recently granted in SCLC and MPM (in combination with chemotherapy and different ICIs), results remain somewhat modest and limited to a small proportion of patients. This work reviews the trial results of ICI therapy in mesothelioma, SCLC, and TETs and discusses the potential of combining ICIs with old drugs.

## Introduction

In the last decade, the treatment landscape of non-small cell lung cancer (NSCLC) changed dramatically, due to the introduction of targeted therapies and immunotherapy. The success of immune checkpoint inhibitor (ICI) therapy in non-oncogene addicted NSCLC [1–4] has led to investigation of these drugs into other thoracic malignancies such as small cell lung cancer (SCLC), malignant pleural mesothelioma (MPM), and thymic epithelial tumors (TETs).

Clinical trials testing immunotherapy in patients with these thoracic cancers have been conducted or are ongoing [5–7]. Unlike NSCLC, where ICIs have represented a breakdown in the treatment armamentarium and have received approval in different settings of disease [8–10], the improvement in outcomes with ICI therapy in rarer thoracic tumors has been somewhat modest and limited to a small proportion of patients [11].

The peculiar pathogenesis underneath these tumors, along with multiple biases in the design of clinical trials were most probably responsible for delaying the availability of effective immunotherapies. Similar to NSCLC, there might be a subgroup of patients more likely to benefit from ICIs [12], but relevant biomarkers have not been determined yet. In addition, lack of funding for clinical research in rare cancer entities such as MPM and TETs has probably precluded the conduction of robust clinical trials that could address specific research questions [13].

What emerges from these recent approvals and from the disappointing results of single-agent ICIs, is the need to properly combine ICIs with other drugs in order to overcome resistance, improve clinical activity and better handle side effects of ICIs in both SCLC, MPM and TETs.

**Figure 1.**
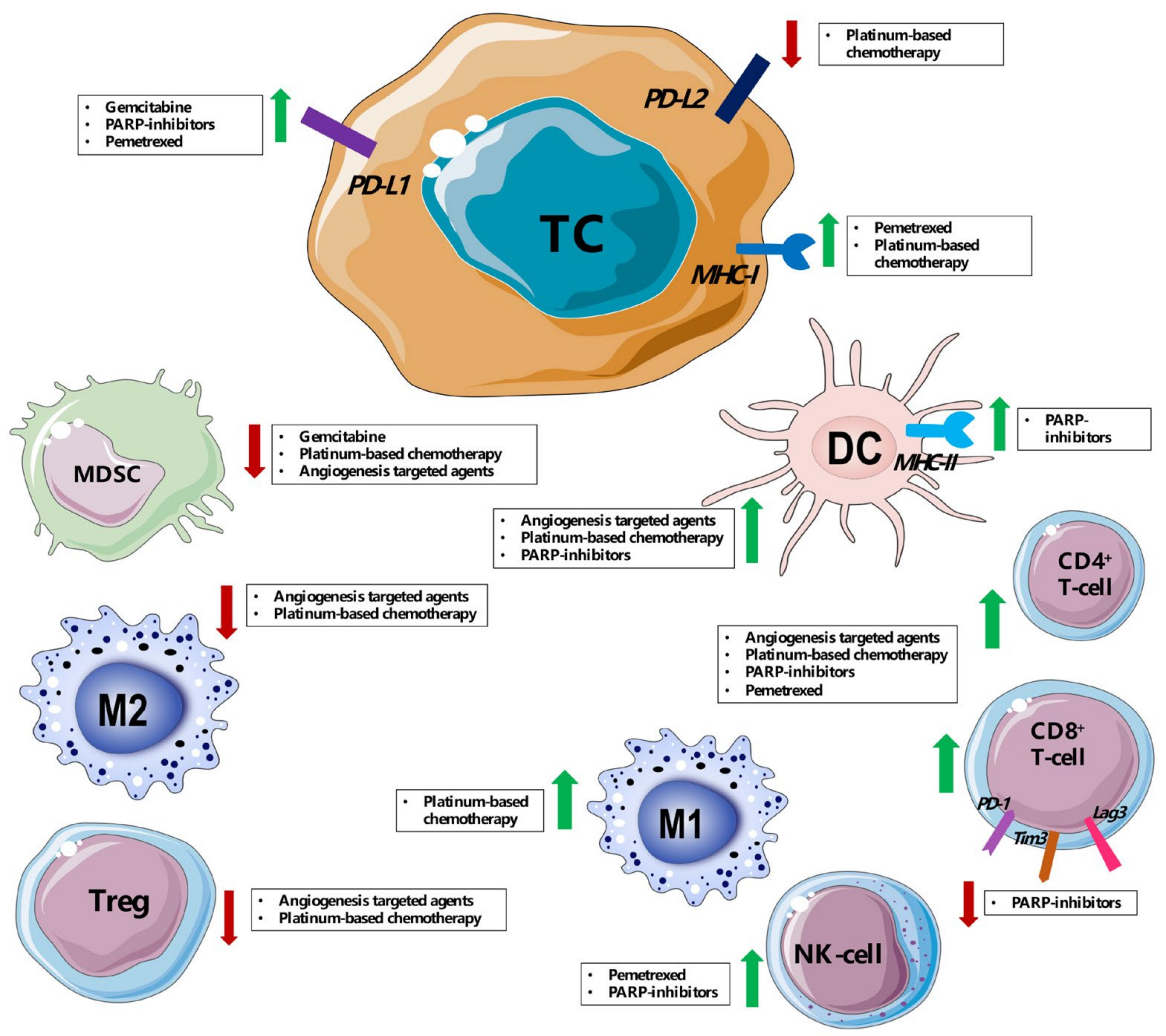
Immune modulation of tumor microenvironment mediated by main drugs [platinum-based chemotherapy, pemetrexed, angiogenesis targeted agents (such as bevacizumab and sunitinib), poly (ADP-ribose) polymerase (PARP) inhibitors] used in combination with ICIs in pre-clinical works and clinical trials for SCLC, MPM and thymic cancer. PD-L2: programmed death-ligand 2; DC: dendritic cell; TC: tumor cell; MHC-I: major histocompatibility complex class I; MHC-II: major histocompatibility complex class II; NK-cell: natural killer cell; MDSC: myeloid-derived suppressor cell; Tregs: regulatory T cells

Other conventional therapies such as chemotherapy and targeted therapy, even the ones with different oncological indications, or more recently developed immunotherapies might synergize with ICIs to counteract the immunosuppressive environment of these tumors (Figure 1).

In this review, we discuss the current role of ICI treatment in SCLC, MPM and TETs, by examining the results coming from already published clinical trials and explore future perspectives for novel combination therapies that could improve ICIs efficacy (Tables 1–3), by discussing their biological rationale as well as the importance of properly selecting patients for every treatment.

**Table 1.** Ongoing trials investigating ICIs in SCLC (Source: Clinicaltrials.gov)

**Combination**	**Treatment**	**Identifier/reference**	**Phase**	**Setting/line of treatment**	**Status**	**Estimated enrollment**	**Notes**
Vaccine	Ipilimumab, nivolumab plus DCs	NCT03406715	II	Recurrent/relapsed	Recruiting	41	Open label, single group assignment
	Adenovirus-transfected autologous DC vaccine plus CIK cells	NCT02688673	I/II	First line	Unknown	30	Open label, single group assignment
	Galinpepimut-S plus pembrolizumab	NCT03761914	I/II	Second line	Recruiting	90	Non-randomized, open label, non-comparative, multicenter, multi-arm study
	Atezolizumab plus autologus DC vaccine	NCT04487756	Ib/II	Maintenance/consolidation	Not yet recruiting	20	Single arm, multicenter, open label, with translational sub-study
	Neoantigen-primed DC vaccines	NCT03871205	I	Recurrent/relapsed	Not yet recruiting	30	Open label, single group assignment
Single agent	Atezolizumab	NCT03059667	II	Recurrent/relapsed	Active, not recruiting	70	Open label, randomized, parallel assignment
	Pembrolizumab	NCT03526887	II	Recurrent/relapsed	Recruiting	110	Open label, non-randomized, multicenter exploratory
	RO7121661	NCT03708328	I	Recurrent/relapsed	Recruiting	280	Open label, multicenter, non-randomized, sequential assignment
	Atezolizumab	NCT03540420	II	Maintenance/consolidation	Recruiting	212	Open label, randomized, parallel assignment
	PD-1 inhibitor JS-001	NCT03971214	I	Maintenance/consolidation	Not yet recruiting	6	Open label, single group assignment, pilot study
	Sintilimab after 4–6 cycles of platinum-etoposide combined with CIK cells	NCT03983759	II	Maintenance/consolidation	Recruiting	40	Open label, single group assignment
ICI	Nivolumab plus ipilimumab	NCT03670056	II	Recurrent/relapsed	Recruiting	40	Open label, single group assignment, pilot study
	ONC-392 plus pembrolizumab	NCT04140526	I	Recurrent/relapsed	Recruiting	91	Open label, non-randomized, sequential assignment
	N-803 plus nivolumab	NCT03228667	IIb	Recurrent/relapsed	Recruiting	636	Open label, parallel assignment, multicohort
	Plinabulin plus nivolumab and ipilimumab	NCT03575793	I/II	Recurrent/relapsed	Recruiting	55	Open label, randomized, parallel assignment
	FT500 plus nivolumab, pembrolizumab, atezolizumab, cyclophosphamide, fludarabine	NCT03841110	I	Recurrent/relapsed	Recruiting	76	Open label, non-randomized, parallel assignment
	AMG 757 plus pembrolizumab	NCT03319940	I	Recurrent/relapsed	Recruiting	212	Open label, randomized, parallel assignment, ascending, multiple dose
	Avelumab plus utomilumab, PF-04518600, PD 0360324, CMP-001	NCT02554812	Ib/II	Recurrent/relapsed	Recruiting	620	Open label, randomized
	Nivolumab plus ipilimumab	NCT02538666	III	Maintenance/consolidation	Active, not recruiting	1,212	Double-blind, randomized, multicenter, parallel assignment
Chemotherapy	Durvalumab plus tremelimumab with chemotherapy	NCT03963414	I	First line	Recruiting	18	Open label, non-randomized, sequential assignment
	Nivolumab plus irinotecan	NCT04173325	I	Recurrent/relapsed	Recruiting	10	Open label, single group assignment
	SHR-1210 plus epirubicin	NCT03755115	II	Recurrent/relapsed	Not yet recruiting	40	Open label, Single group assignment
	Avelumab plus platinum and etoposide	NCT03568097	II	First line	Recruiting	55	Open label, single group assignment
	HLX10 plus carboplatin and etoposide	NCT04063163	III	First line	Recruiting	489	Double-bind, randomized, multicenter
	Carillizumab plus apatinib, etoposide and cisplatin	NCT04490421	III	First line	Not yet recruiting	45	Open label, single group assignment
	Camrelizumab, apatinib, irinotecan plus platinum	NCT04453930	II	First line	Recruiting	60	Open label, single group assignment
	Atezolizumab plus carboplatin and etoposide	NCT04221529	II	First line	Recruiting	70	Open label, single group assignment
	BGB-A317 plus cisplatin and etoposide	NCT04542369	II	Neoadjuvant/first line	Not yet recruiting	15	Open label, single group assignment
	TQB2450 plus cisplatin and etoposide	NCT04539977	II	First line	Not yet recruiting	40	Open label, non-randomized, sequential assignment
	Durvalumab plus carboplatin and etoposide	NCT04472949	II	Maintenance/consolidation	Not yet recruiting	46	Open label, prospective, multicenter, single group assignment
	Nivolumab plus cisplatin/carboplatin plus etoposide	NCT03382561	II	First line	Active, not recruiting	150	Open label, randomized, parallel assignment
	Protein phosphatase 2A inhibitor LB-100, atezolizumab, carboplatin and etoposide	NCT04560972	Ib	First line	Not yet recruiting	18	Open label, single group assignment
	M7824 plus topotecan or temozolomide	NCT03554473	I/II	Recurrent/relapsed	Recruiting	67	Open label, non-randomized, sequential assignment
	Nivolumab plus temozolomide	NCT03728361	II	Recurrent/relapsed	Recruiting	53	Open label, single group assignment
	RRx-001 plus cisplatin/carboplatin and etoposide	NCT03699956	III	Third line	Active, not recruiting	126	Open label, randomized, crossover assignment
	ZKAB001 plus carboplatin and etoposide	NCT04346914	I	First line	Recruiting	20	Open label, single group assignment
	Pambrolizumab plus cisplatin/carboplatin and etoposide	NCT02934503	II	First line	Recruiting	NA	Open label, single group assignment
	Atezolizumab plus carboplatin and etoposide	NCT02763579	III	First line	Active, not recruiting	403	Double blind, randomized, placebo-controlled
	Pembrolizumab plus cisplatin/carboplatin and etoposide	NCT03066778	III	First line	Active, not recruiting	453	Double blind, randomized, placebo-controlled
	PM01183 plus atezolizumab	NCT04253145	I/II	Recurrent/relapsed	Recruiting	25	Open label, prospective, uncontrolled and multicenter, single group assignment
Radiotherapy	Atezolizumab plus radiotherapy	NCT04402788	II/III	Maintenance/consolidation	Recruiting	324	Open label, randomized, parallel assignment
	Ipilimumab, nivolumab plus radiotherapy	NCT03043599	I/II	Maintenance/consolidation	Active, not recruiting	21	Open label, single group assignment
	CS1001 plus radiotherapy	NCT04421352	I/II	Maintenance/consolidation	Not yet recruiting	20	Open label, single group assignment
	177Lu-DOTA0-Tyr3-Octreotate, nivolumab	NCT03325816	I/II	Maintenance/consolidation	Active, not recruiting	9	Open label, randomized, sequential assignment
Chemoradiotherapy	Durvalumab+/-tremelimumab plus platinum/etoposide plus radiotherapy (standard *vs*. hyperfractionated)	NCT03509012	I	First line	Active, not recruiting	105	Open label, non-randomized, parallel assignment, multicenter
	SHR1316, carboplatin, etoposide, radiotherapy	NCT04562337	II	Maintenance/consolidation	Not yet recruiting	67	Open label, single group assignment
	Pembrolizumab, cisplatin/carboplatin, etoposide and radiotherapy	NCT02402920	I	First line	Recruiting	84	Open label, non-randomized, parallel assignment
	Atezolizumab, cisplatin/carboplatin, etoposide and radiotherapy	NCT03811002	II/III	First line	Recruiting	506	Open label, randomized, parallel assignment
	Sintilimab, cisplatin, etoposide and radiotherapy	NCT04189094	II	First line	Not yet recruiting	140	Open label, randomized, parallel assignment
Anti-angiogenic	Anlotinib plus sintilimab	NCT04192682	II/III	Second line	Recruiting	40	Open label, single group assignment
	Anlotinib plus sintilimab	NCT04055792	II	Third line	Recruiting	52	Randomized, controlled, parallel assignment
	TQB2450, anlotinib, carboplatin and etoposide	NCT04234607	III	First line	Not yet recruiting	738	Double-blind, randomized, controlled, multicenter
	Vorolanib plus nivolumab	NCT03583086	I/II	Recurrent/relapsed	Recruiting	177	Open label, single group assignment
	Cabozantinib S-malate plus nivolumab	NCT04514484	I	Recurrent/relapsed	Not yet recruiting	18	Open label, single group assignment, pilot study
	Anlotinib plus PD-1/L1 inhibitor	NCT04313660	II	Maintenance/consolidation	Not yet recruiting	33	Open label, single group assignment
	SHR-1210 plus apatinib	NCT03417895	II	Recurrent/relapsed	Unknown	135	Open label, randomized, parallel assignment
	Anlotinib plus durvalumab	NCT04314297	II	Maintenance/consolidation	Not yet recruiting	33	Open label, single group assignment
	Surufatinib plus tislelizumab	NCT04579757	Ib/II	Recurrent/relapsed	Not yet recruiting	120	Open label, non-randomized, sequential assignment
PARP inhibitors	Atezolizumab plus talazoparib	NCT04334941	II	Maintenance/consolidation	Recruiting	94	Open label, randomized, parallel assignment
	Durvalumab plus olaparib	NCT02734004	I/II	Recurrent/relapsed	Active, not recruiting	427	Open label, single group assignment
	Rucaparib plus nivolumab	NCT03958045	II	Maintenance/consolidation	Recruiting	36	Open label, single group assignment
	Talazoparib, pembrolizumab and ZN-c3	NCT04158336	I/II	Recurrent/relapsed	Recruiting	360	Open label, non-randomized, parallel assignment
Other	Sintilimab plus metformin	NCT03994744	II	Recurrent/relapsed	Recruiting	68	Open label, single group assignment
	AZD8701 plus durvalumab	NCT04504669	I	Recurrent/relapsed	Recruiting	123	Open label, non-randomized, sequential assignment
	GB1275 plus pembrolizumab	NCT04060342	I/II	Recurrent/relapsed	Recruiting	242	Open label, non-randomized, sequential assignment
	RGX-104 plus nivolumab, ipilimumab, docetaxel, pembrolizumab, carboplatin, pemetrexed	NCT02922764	I	Recurrent/relapsed	Recruiting	135	Open label, non-randomized, parallel assignment
	Pembrolizumab plus NT-I7	NCT04332653	I/II	Recurrent/relapsed	Recruiting	168	Open label, non-randomized, sequential assignment

CIK: cytokine-induced killer; PD-1: programmed cell death protein 1; NA: not available

**Table 2.** Ongoing trials investigating ICIs in MPM (Source: Clinicaltrials.gov)

**Combination**	**Treatment**	**Identifier/reference**	**Phase**	**Setting/line of treatment**	**Status**	**Estimated enrollment**	**Notes**
Vaccine	Nivolumab plus galinpepimut-S	NCT04040231	I	Reccurent/relapsed	Recruiting	10	Open label, single group assignment
Single agent	Nivolumab	NCT03063450 (CONFIRM)	III	Relapsed	Active, not recruiting	336	Double-blind, placebo controlled
ICI	Nivolumab plus ipilimumab	NCT02899299 (CheckMate-743)	III	First Line	Active, not recruiting	606	Open label, randomized
	Nivolumab/nivolumab plus ipilimumab	NCT02716272 (MAPS2)	II	Relapsed	Active, not recruiting	125	Open label, randomized
Chemotherapy	Durvalumab plus chemotherapy	NCT02899195 (PrE0505)	II	First line	Active, not recruiting	55	Open label, non-randomized
	Pembrolizumab/pembrolizumab plus chemotherapy	NCT02784171	II/III	First line	Active, not recruiting	520	Open label, randomized
	Nivolumab plus chemotherapy	NCT04177953	II	Adjuvant	Recruiting	92	Open label, randomized
	Atezolizumab plus gemcitabine	NCT04480372	II	Relapsed	Not yet recruiting	67	Open label, single group assignment
	Atezolizumab plus cisplatin and pemetrexed	NCT03228537	I	Neoadjuvant	Active, not recruiting	28	Open label, single group assignment
Radiotherapy	Pembrolizumab after radiation therapy	NCT02959463	I	Maintenance	Recruiting	24	Open label, non-randomized
Anti-angiogenic	Atezolizumab plus bevacizumab and chemotherapy	NCT03762018 (BEAT-meso)	III	First line	Recruiting	320	Open label, randomized
Photodynamic therapy	Nivolumab after intrapleural photodynamic therapy	NCT04400539	II	Maintenance	Not yet recruiting	20	Open label, single group assignment, pilot study

**Table 3.** Ongoing trials investigating ICIs in thymic epithelial cancer patients (Source: Clinicaltrials.gov)

**Combination**	**Treatment**	**Identifier/reference**	**Phase**	**Setting/line of treatment**	**Status**	**Estimated enrollment**	**Notes**
Chemotherapy	Chemotherapy plus pembrolizumab	NCT04554524	II	First line	Recruiting	40	Open label, single group assignment
Single agent	Pembrolizumab	NCT02607631	II	Recurrent	Completed	33	Open label, single group assignment
	Nivolumab	NCT03134118	II	Recurrent	Recruiting	55	Open label, single group assignment
	Avelumab	NCT03076554	II	Recurrent	Recruiting	55	Open label, single group assignment
	Atezolizumab	NCT04321330	II	Recurrent	Recruiting	34	Open label, single group assignment
Radiotherapy	Pembrolizumab, docetaxel, cisplatin therapy followed by surgery/radiotherapy plus pembrolizumab	NCT03858582	II	Neo-adjuvant	Not yet recruiting	40	Non-randomized
Anti-angiogenic	Pembrolizumab plus sunitinib	NCT03463460	II	First line	Recruiting	40	Open label, single group assignment
	Nivolumab plus vorolanib	NCT03583086	I/II	First line	Recruiting	177	Open label, single group assignment. thoracic tumors including thymic cancer

## SCLC

Around 15% of lung cancers is represented by SCLC, a neuroendocrine tumor characterized by a high growth fraction and an early development of distant metastasis. SCLC can be staged using both the American Joint Committee on Cancer (AJCC) TNM and the historical Veterans Affairs (VA) classification system, which has only 2 stages: limited (disease confined to the ipsilateral hemithorax, which could be included in a radiation therapy filed) and extensive (anything beyond limited stage). The clinical implication of VA system made it extremely useful even today in clinical practice as well as research.

SCLC is strictly related to tobacco use. Smoking is known to damage DNA [14] and consequently determine a high quantity of somatic mutations, the so-called as tumor mutation burden (TMB), that is a well-documented biomarker for immunotherapy [15, 16]. This feature can lead to a release of tumor neoantigens capable to stimulate an anti-tumoral immune response, thus representing the rationale for immunotherapy activity in this disease [17].

Unfortunately, ICIs showed only moderate benefit when investigated in SCLC. Although the phase I/II CheckMate-032 trial assessing nivolumab with or without ipilimumab in relapsed SCLC documented a 1-year overall survival (OS) of 42% for nivolumab/ipilimumab combination and 30% for nivolumab alone [18], the phase III CheckMate-331 reported that nivolumab was not superior to topotecan in patients with relapsed or progressed SCLC after a platinum-based treatment in terms of OS, progression-free survival (PFS) and objective response rate (ORR) [19]. Notably, a late separation of the curves and a potential activity in the platinum-refractory setting suggests a possible long-term benefit for a subgroup of patients.

The global, double-blind, phase III study compared nivolumab plus ipilimumab or nivolumab alone *vs*. placebo as maintenance therapy in patients with extensive SCLC who did not progress to first line platinum chemotherapy. Both nivolumab plus ipilimumab and nivolumab alone did not improve OS compare to placebo but maintenance immunotherapy appeared to improve PFS, with rates of patients who were progression-free at six months of 20% and 21% for nivolumab with or without ipilimumab, respectively, *versus* 10% for placebo [20].

The KEYNOTE-028 and the KEYNOTE-158 trials documented a prolonged durable response for SCLC patients after 2 or more lines [21]. However, these two were a phase Ib and a phase II studies, respectively, and a large randomized controlled trial with pembrolizumab in relapsed SCLC is missing.

Noteworthy, due to the paucity of therapeutic alternatives, nivolumab and pembrolizumab got the FDA approval for the third or later line treatment for SCLC, based on CheckMate-032 and KEYNOTE-028/KEYNOTE-158 trial results. In CheckMate-032 PD-L1 staining seems to not be a predictor of response to nivolumab [22]. Exploratory analysis from the KEYNOTE-158 suggested a role of PD-L1 expression in patients with SCLC who may response to pembrolizumab [21]. Both in CheckMate-032 and in KEYNOTE-158 patients characterized by a high TMB seems to better response to nivolumab or pembrolizumab but further studies are needed to identify new biomarkers of response.

Different mechanisms have been proposed to justify the poor effectiveness of immunotherapy in this disease, such as low PD-L1 expression, downregulation of MHC molecules, immunosuppression induced by SCLC cells and autocrine and paracrine regulation [23, 24].

Therefore, further treatment strategies are required to overcome these mechanisms of ICI resistance, and drug combinations seem to be a promising approach.

### Chemotherapy + ICIs

Historically, first-line treatment in SCLC has been represented by chemotherapy with platinum (cisplatin or carboplatin) plus etoposide, which showed a good ORR of about 65%, but an OS far less satisfying, around 9–10 months [25].

In NSCLC, the combination of chemotherapy and immunotherapy has become the new standard of care in first-line, with exciting results [26, 27]. The rationale behind this synergism is the immunogenic cell death (ICD) induced by cytotoxic therapy and the concurrent appearance of specific damage-associated molecular patterns (DAMPs) on the surface of the apoptotic cells, able to trigger an anti-tumor immune response by the promotion of DC maturation and activation of cytotoxic T lymphocytes (CTLs). Meanwhile, chemotherapy is thought to reduce the immunosuppressive activity of tumor microenvironment, specially downregulating the MDSCs [28, 29].

Using chemotherapy is even clinically more necessary in SCLC, being a rapidly progressive disease, which requires a rapid tumor shrinkage, therefore a monotherapy with ICIs may be too ineffective. Until now, two studies were published showing efficacy of chemotherapy combined with ICIs and imposing a new standard of treatment in the first-line setting of ES-SCLC.

In the phase III trial IMpower133, atezolizumab was administered with carboplatin and etoposide in patients with ES-SCLC who were not previously treated [5]. The study documented a significant prolonged OS and PFS for patients who received immunotherapy compared to those treated with chemotherapy plus placebo [median OS (mOS) 12.3 months *vs*. 10.3 months, *P* = 0.007 with HR of 0.70 and median PFS (mPFS) 5.2 months *vs*. 4.3 months, *P* = 0.02 with HR of 0.77], leading to a gain of 13.5% of patients alive at 1 year. ORR did not differ between the two treatments.

The second phase III trial showing benefit in adding immunotherapy to standard treatment as first-line in patients with ES-SCLC was the CASPIAN trial [6]. The trial had also an arm in which an anti-cytotoxic T-lymphocyte-associated protein 4 (CTLA4) was added (durvalumab plus platinum-etoposide regimen, durvalumab plus tremelimumab plus chemotherapy and the chemotherapy control arm), but the planned interim analysis did not include the tremelimumab arm.

The results were in line with those of the IMpower133. In fact, adding durvalumab (an anti-PD-L1 agent) to standard chemotherapy showed to significantly improve OS compared to control arm, 13.0 months *vs*. 10.3 months (*P* = 0.0047 with HR of 0.73) with 34% *vs*. 28% patients alive at 18 months, respectively. Recently, the trial update confirmed the benefit in OS (12.9 months *vs*. 10.5 months, *P* = 0.0032 with HR of 0.75, after a median follow up of 25.1 months). However, the study’s third arm with tremelimumab did not meet the prespecified threshold for statistical significance (*P* ≤ 0.0418). Indeed, the median OS for this combination was 10.4 months *vs*. 10.5 months for control arm (*P* = 0.0451, HR 0.82) and the 18-months OS rates was 32.0% in the durvalumab + chemotherapy, 30.7% in the tremelimumab + durvalumab + chemotherapy, and 24.8% in the control arm; at 24 months, those rates were 22.2%, 23.4%, and 14.4%, respectively. Therefore, the only combination available in first-line remains without the CTLA4 inhibitor.

Although similar, the two studies have few differences such as the number of cycles of platinum-etoposide in the control group (4 for IMpower133 and 6 for CASPIAN) that could have influenced the outcome and the possibility of investigator’s choice of platinum, which was possible in CASPIAN but not in IMpower133 (where only carboplatin was allowed). Interestingly, the last update of CASPIAN reported that durvalumab was associated with prolonged OS regardless of which platinum was administered.

Furthermore, two more studies investigated the association of standard platinum-etoposide chemotherapy with pembrolizumab, the phase III KEYNOTE-604 trial, or with nivolumab, the phase II ECOG-ACRIN EA5161. In the KEYNOTE-604, at final analysis (median follow up 21.6 months), 9% of patients in the pembrolizumab arm and 1% in the control arm were still on treatment. In the intention-to-treat (ITT) population, the combination prolonged OS, but did not meet the prespecified significance threshold of *P* = 0.0128 (*P* = 0.0164 with HR of 0.80, median OS 10.8 months *vs*. 9.8 months with or without pembrolizumab respectively), thus making the results of this study not as robust as the others with different ICIs [30]. Differently, the association of platinum-etoposide chemotherapy with nivolumab improved both the median OS and the mPFS compared to chemotherapy in the ITT population (median OS 11.3 months *vs*. 8.5 months, *P* = 0.038 with HR of 0.67 and mPFS 5.5 months *vs*. 4.6 months, *P* = 0.012 with HR of 0.65), giving a potential ICI alternative to atezolizumab and durvalumab.

Besse et al. [31] at ESMO 2020 presented the results of REACTION study, a multicenter open-label randomized phase II trial. They randomized patients with extensive SCLC, unselected for PD-L1, and controlled brain metastases who detained an objective response after 2 cycles of chemotherapy with platinum and etoposide to receive pembrolizumab in combination with four additional cycles of platinum etoposide then pembrolizumab up to 35 cycles (experimental arm) *vs*. four additional platinum etoposide cycles (control arm). Even though pembrolizumab combined with platinum and etoposide did not improve PFS, data showed benefit in OS [mOS was 12.3 months for the experimental arm and 10.4 for control arm (*P* = 0.097, one-sided)], suggesting a potential role of pembrolizumab in first line treatment to improve chemotherapy efficacy.

Reck et al. [32], investigated the combination of ipilimumab plus etoposide and platinum *vs*. placebo plus etoposide and platinum in a phase III randomized trial including patients affected by extensive-stage disease SCLC. The addiction of ipilimumab to chemotherapy did not show significant improvement both in OS and in the other secondary endpoints (PFS, ORR).

In addition to studying different ICIs, changing chemotherapy backbone is also a possible strategy that is worth to investigate. Anthracyclines, for example, represent a potential therapeutic strategy for SCLC treatment in combination with cyclophosphamide, adriamycin and vincristine (CAV regimen) [33]. It is well documented their activity both in first-line, before cisplatin/etoposide therapy showed to be superior [34], and in second-line, where no differences in terms of ORR (18.3% *vs*. 24%, *P* = 0.285), median time to progression (12.3 weeks *vs*. 13.3 weeks, *P* = 0.552) and mOS (24.7 weeks *vs*. 25.0 weeks, *P* = 0.795) with topotecan were found [35].

Moreover, it was demonstrated that anthracyclines, in particular doxorubicin, enhance an anticancer-immune activity by inducing calreticulin (CRT) exposure on dying cancer cell, which is a fundamental step for ICD mediated by DCs. Interestingly, the same mechanism was not documented in etoposide-treated mice [36]. Therefore, it is possible to hypothesize that immunotherapy with different chemotherapy-combination, based on doxorubicin rather than etoposide for example, could lead to more promising results [37].

A novel drug recently designed as orphan drug to treat patients with relapsed SCLC is lurbinectedin, that induces apoptosis by inhibition of RNA polymerase II through blocking trans-activated transcription. Lurbinectedin has demonstrated to be active as a single agent, in a phase II basket trial, as well as in combination with doxorubicin in a phase Ib, especially in patients with chemotherapy-free interval of 90 or more days, showing an ORR of 53% and a PFS of 5.7 months [38, 39].

Based on these encouraging results, a prospective, open-label, uncontrolled and multicenter phase I/II study of lurbinectedin in combination with pembrolizumab in patients with relapsed SCLC is ongoing (NCT04358237).

To conclude, although the clinical impact of the studies reported above might be not so impressive compared to those achieved in other malignancies, these results further confirm that combining chemotherapy with ICIs could be a promising treatment option, and different ICIs as well as different chemotherapies need to be evaluated to find the most efficient association.

### DNA damage response (DDR) inhibitors + ICIs

Another combination which is stimulating the scientific community is immunotherapy with DDR inhibitors. It has been demonstrated in breast cancer and NSCLC that treatment with PARP inhibitors enhances expression of PD-L1 by increasing cytosolic DNA and consequently activating the cyclic GMP-AMP synthase (CGAS)/stimulator of interferon genes (*STING*) innate immune signaling. The entire process stimulates PD-L1 expression and T cell recruitment [40–42].

A similar pathway has been described for SCLC. A pre-clinical study observed in murine models an increase of PD-L1 expression and T-cytotoxic infiltration, decreased T-cell exhaustion and tumor shrinkage when either olaparib (a PARP inhibitor) or prexasertib (a checkpoint kinase 1 inhibitor) were associated to an anti-PD-L1 agent [43]. On the contrary, the MEDIOLA trial, a phase II basket trial studying the association of olaparib and durvalumab in different relapsed cancers, did not observe a clinical efficacy in SCLC unlike other tumors, such as breast or ovarian cancers [44]. Among 38 patients with ES-SCLC, only 4 had a tumor response, according to RECIST criteria, and 11 experienced disease control. In line with this result, a single arm, phase II study documented a partial or complete response in only 2 out 19 patients, who started at the same time durvalumab and olaparib (rather than adding durvalumab to the PARP inhibitor afterwards as in the MEDIOLA trial) [45].

Nevertheless, some considerations have to be done before considering this combination a failure. Firstly, platinum and PARP1 resistance are correlated, and because both trials were conducted in patients with platinum resistance/refractory disease, it could be reasonable to speculate that the combination may be more active before the resistance is developed. Secondly, germline *BRCA1* or *BRCA2*-mutation (which are well-known biomarker of efficacy for PARP inhibitor in other malignancies) are very rare in SCLC, thus probably explaining a minor activity of this combination. Patient selection should be rather based on the expression of schlafen family member 11 protein (SLFN11), which is more often documented in SCLC and also related to PARP1 sensitivity [46, 47]. Thirdly, patients who experienced response in both studies reported above had an inflamed phenotype (i.e. CD8-positive T-cells in direct contact with tumor) or PD-L1-positive staining within tumor cells. Therefore, selecting patients by clinical outcome, biomarkers or immune phenotypes might allow this combination to be more effective and useful.

### Angiogenesis targeted agents + ICIs

Pro-angiogenic factors impact the immune surveillance in different ways, by suppressing the function of several immune cells [48], decreasing the leukocyte-endothelial interactions and hampering the infiltration of immune effector cells into the tumor microenvironment [49]. It has been shown that maturation of DC precursors is suppressed by high vascular endothelial growth factor (VEGF) levels [50]. On the contrary, proliferation of immune suppressive cells such as Tregs cells and immature myeloid cells, is promoted by high VEGF levels [51]. The SCLC tumor tissue expresses high levels of VEGFA, VEGF receptors and PD-L1 [52, 53]. A pre-clinical work analyzed the efficacy of an anti-PD-L1 and anti-VEGF drug combination in a mouse model of SCLC, showing a synergic positive effect. The study showed how ICIs combined with anti-VEGF modified the tumor-infiltrating T cells. In fact, on mice not treated, T cells were not present on the pulmonary tissue around the tumor and on the tumor infiltration, whereas on anti-VEGF treated mice CD4^+^ T cells infiltrated tumor, with CD8^+^ at tumor margin. In addition, on mice treated with only anti-PD-L1, CD4^+^ accumulated in tumor margin without invading tumor tissue; only on mice treated with combination of anti-VEGF and anti-PD-L1 it was possible to see CD4^+^ T cells and CD8^+^ T cells infiltrate tumor tissue. The results strongly recommend a combination therapy of anti-VEGF agents and ICIs for the treatment of patients with SCLC [54].

During last years, receptor tyrosin kinases (RTKs) have become the target of several drugs used in clinical practice in NSCLC [55], but no survival advantages were obtained from clinical trials that investigated tyrosine kinase inhibitors (TKIs) in SCLC patients. Cabozantinib is a small-molecule kinase inhibitor, with activity toward MET, VEGF receptors 2 (VEGFR2) and other tyrosine kinases like RET, KIT, AXL, and FLT3 [56]. Due to the inhibitory effect of cabozantinib on the VEGFR2, it might promote the reprogramming of the immunosuppressive tumor microenvironment and synergize with ICIs [51]. On this basis, an ongoing phase II trial is investigating the combination of cabozantinib, nivolumab and ipilimumab in patients with poorly differentiated neuroendocrine tumors (NCT04079712). Another phase I/II dose escalation and dose expansion trial of combination of oral vorolanib, a VEGFR/PDGFR dual kinase inhibitor, and nivolumab is ongoing in patients with NSCLC, SCLC and thymic carcinomas (TC) (NCT03583086).

In conclusion, since the impact of ICI monotherapy on SCLC is limited, further combination strategies with drugs able to modulate the tumor immune microenvironment may become a stronger weapon to improve SCLC patient outcome [57].

## MPM

MPM is a rare and aggressive tumor arising from mesothelial cells of the pleura most commonly caused by mineral fibers (such as asbestos and erionite) exposure [58].

Even though surgery and radiotherapy play a role in this pathology, the current established therapy is still systemic chemotherapy with cisplatin and pemetrexed on the basis of the phase III EMPACHIS trial which demonstrated a 3-months survival benefit compared to cisplatin alone (12.1 months *vs*. 9.3 months) [59, 60].

The limited benefit of first-line chemotherapy and the lack of an effective second-line strategy, with exception of the reintroduction of a pemetrexed-based chemotherapy in patients with durable response to front-line chemotherapy [60] prompt the detection of new therapeutic strategies.

ICIs have been an active field of research in MPM both for their successes in other malignancies and the important role which immune system exerts in the pathogenesis of MPM [61]. Some cases of spontaneous MPM regression likely related to an activation of the immune system have been reported [62] and a worse outcome has been related with high CD163^+^ tumor-associated macrophages and low CD8^+^ tumor infiltrating lymphocytes [63, 64]. Furthermore PD-L1 was shown to be highly expressed in MPM cells and PD-L1 positivity has proven to be an independent risk factor for survival in MPM patients [65].

Therefore, various PD-1/PD-L1 inhibitors have been investigated in patients with disease progression after first-line chemotherapy. In a phase II trial pembrolizumab showed a disease control rate (DCR) of 47% with a PFS of 4.5 months [66] while, in two phase II trials investigating single agent nivolumab, the PFS was 2.6 and 6.1 months, respectively [67, 68]. Similar results were reported for avelumab [69].

A randomized study comparing pembrolizumab with chemotherapy (gemcitabine or vinorelbine) in MPM patients with disease recurrence reported almost equal PFS (2.5 *vs*. 3.4) and OS (10.7 *vs*. 11.7) between the two groups [70]. A phase III trial (CONFIRM trial) with randomization to nivolumab or placebo in MPM patients which progressed after at least 2 chemotherapy lines is currently ongoing [71].

Despite initial promising results in phase II trials [72, 73] tremelimumab, a human antibody against CTLA4, failed to demonstrate a benefit compared to placebo in a large randomized trial (mOS of 7.7 months *vs*. 7.3 months, respectively) [74].

The disappointing results of ICI monotherapy could be due both to the lack of biomarkers able to identify the proper candidate for immunotherapy, since PD-L1 expression seems not to be a reliable prognostic tool [75] and to the immunogenic characteristics of MPM. The limited mutation rate and the resultant low formation of antigens [76, 77] together with the potential upregulation of different inhibitory checkpoints, such as TIM-3 and LAG-3, resulting from anti-PD-1/PD-L1 and anti-CTLA4 drugs use [78], might explain the limited efficacy of ICIs.

### Double immune checkpoint inhibition

In order to overcome these obstacles, the combination of two ICIs targeting different inhibitory checkpoints or the association of ICIs with chemotherapy might be a successful strategy.

The inhibition of PD-1 pathway, primarily involved in effector T-cell and NK-cells inhibition in peripheral tissue, and the blockade of CTLA4, which plays an important role in lymph nodes T-cell activation and in DCs suppression [79], demonstrated a synergistic effect in different types of cancer [80–82].

The phase II MAPS2 trial randomized patients to nivolumab or the combination of nivolumab and ipilimumab achieving the primary endpoint in both arms (DCR of 44.4% and 50% respectively). The survival analysis demonstrated a mOS 11.9 months in the nivolumab arm and 15.9 months for the combination arm [83]. On the basis of this trial, the FDA gave orphan drug designation to nivolumab or nivolumab plus ipilimumab in MPM patients after progression to first-line therapy.

The combination of tremelimumab and durvalumab was investigated in a phase II trial (NIBIT-MESO-1) as first and second-line treatment. This study enrolled patients with malignant pleural and peritoneal MPM and showed an ORR of 28% (reaching its primary endpoint), a DCR of 65%, a mPFS of 8 months and a mOS of 16.6 months [84].

Similarly, the INITIATE trial investigated the activity of nivolumab with ipilimumab after first line treatment in MPM and peritoneal MPM patients and achieved the primary endpoint with DCR of 68% [85].

The results of a prespecified interim analysis of a phase III randomized trial (CheckMate-743) [7] comparing the combination of nivolumab and ipilimumab *vs*. cisplatin/carboplatin and pemetrexed as first-line therapy in unresectable MPM were presented during the 2020 World Conference on Lung Cancer Virtual Presidential Symposium. A total of 605 patients were enrolled and, at a median follow-up of 29.7 months, the mOS was significantly longer for the nivolumab plus ipilimumab arm (18.1 months) than the chemotherapy arm (14.1 months). This study is the first randomized trial which demonstrated the superiority of ICIs combination over chemotherapy in first-line treatment for MPM patients, corroborating the rationale of dual immunotherapy strategy in this setting and leading to the recent approval by the FDA for this combination for first-line treatment in unresectable MPM adult patients.

### Chemotherapy + ICIs

The combination of immunotherapy with chemotherapy is further a viable strategy as evidenced in malignancies including, for instance, breast cancer [86] and NSCLC [26, 27].

Beside the previously described mechanism for SCLC by which chemotherapy can broadly activate the immune system, an *in vitro* study investigated the effect of cisplatin, oxaliplatin and pemetrexed on three different MPM cells lines and highlighted a potential downregulation effect of cisplatin on immune checkpoints expression (PD-1, LAG-3, TIM-3) suggesting that this chemotherapeutic agent might be a promising partner for ICIs also in MPM [87].

The combination of durvalumab with cisplatin and pemetrexed chemotherapy as first-line therapy was investigated in a single arm phase II trial (DREAM study) which reported an ORR of 48% and a mPFS of 6.9 months [88].

The same combination was investigated in a recent phase II trial (PrE0505 study) which enrolled 55 patients with previously untreated unresectable MPM. This trial, presented at the virtual 2020 ASCO Annual Meeting, reached its primary endpoint, with a promising mOS of 20.4 months (*P* = 0.0014, one-sided) [89].

The results of a phase II trial investigating pembrolizumab alone and in combination with cisplatin and pemetrexed in first-line setting are awaited (NCT02784171). The combination of ICI (nivolumab) with platinum-based chemotherapy is under investigation also in the adjuvant setting in a randomized controlled trial which is still recruiting (NCT04177953).

### Angiogenesis targeted agents + ICIs

As previously discussed, tumors can evade immune response through VEGF-induced irregular vascularization and consequential T-cell infiltration hampering [90], suggesting that anti-VEGF agents could represent an ideal partner for ICIs to overcome immune resistance mechanisms.

Furthermore, given that VEGF exert a prominent role in MPM angiogenesis [91], anti-VEGF agents, either alone or in combination with chemotherapy, have been investigated in MPM patients, though with poor results [92–94]. The MAPS trial, a large phase III randomized study, investigated the combination of bevacizumab with cisplatin and pemetrexed, demonstrating a significantly enhanced mOS with the addition of the anti-VEGF agent (18.8 *vs*. 16.1 months, *P* = 0.0167) [95]. This triplet combination regimen can be considered as first-line treatment for MPM but, even if it has been validated by some US and European guidelines, it is not currently approved by the FDA and the European Medicines Agency (EMA) as the MAPS study was not a pivotal trial.

Against this background, the BEAT-meso trial was designed in order to investigate the efficacy of atezolizumab combined with bevacizumab and chemotherapy. In this phase III trial patients with unresectable MPM will be randomly assigned to treatment 1 group (4–6 cycles of carboplatin plus bevacizumab) or treatment 2 group (4–6 cycles of carboplatin plus bevacizumab plus atezolizumab).

This multicentre trial is still recruiting, and its results are awaited as they could further revolutionize the treatment of this orphan disease.

## TETs

TETs are a heterogeneous group of thoracic cancers, with an annual incidence of about 1.3 to 3.2 per million [96]. The World Health Organization classification stratified TETs into A, AB, B1, B2, and B3 thymomas and TC, taking into account characteristics of malignant epithelial cells and the percentage of non-neoplastic lymphocytes [97, 98]. TC represent about 10–12% of TETs and show a more aggressive clinical behavior and worse overall prognosis compared to thymomas [99]. If complete resection is possible due to early stage, surgery represents the first treatment choice. Locally advanced TETs require a multimodality approach characterized by chemotherapy and radiotherapy in addition to surgery. First-line platinum-based chemotherapy should be considered for advanced non-resectable or metastatic TETs [96]. Treatment options for relapsed or refractory disease after first-line are limited. Imatinib, a KIT TKI, may represent a therapy option for patients with TC who have progressed after first-line chemotherapy, in case of detection of *c-KIT* gene activating mutations [100, 101]. Sunitinib, an anti-angiogenic multikinase inhibitor, could be used with the same indication, regardless from KIT status [102]. The thymus detains a key role in the development of immune tolerance and, through complex steps, leads to the development of central T-cell tolerance, necessary to avoid the onset of autoimmune diseases [103]. In the thymic medulla, the autoimmune regulator (*AIRE*) gene and the transcription factor FEZ family zinc finger 2 (FEZF2) promote the expression of tissue-specific antigens (TSAs) in order to develop T-cells. Those cells which react against TSAs undergo apoptosis [104, 105]. About 30% of thymoma patients develop autoimmune disorders, such as myasthenia gravis (the most frequent), pure red cell aplasia, hypogammaglobulinemia, systemic lupus erythematosus and pemphigus [106, 107]. This is due to the downregulation of AIRE and FEZF2, the overthrow of the normal thymic histological architecture, the deficient expression of MHC class II molecules by thymoma cells. These mechanisms lead to the failure of central immune tolerance and susceptibility to auto-immunity [108, 109].

Results obtained from several clinical trials with ICIs as monotherapy are encouraging for TETs showing promising outcome with a response rate (RR) of approximately 20% in pre-treated patient populations [110–113].

Giaccone et al. [110], assessed the activity of pembrolizumab in patients with advanced TC who had progressed after at least one line of chemotherapy, in a single-arm phase 2 study. The results were promising, in fact 22.5% of patients achieved a response; one (3%) patient showed a complete response, eight (20%) patients showed partial responses, and 21 (53%) patients stable disease. Six (15%) patients developed severe autoimmune toxicity, including two (5%) patients with myocarditis. No deaths due to toxicity were observed.

Another phase II study evaluated the efficacy and safety of pembrolizumab in a cohort of 26 patients affected by TC and seven patients affected by thymoma, progressed after at least one line of platinum-based chemotherapy. Of seven thymomas, two (28.6%) obtained partial response, and five (71.6%) obtained stable disease. Of 26 TC, five (19.2%) obtained partial response and 14 (53.8%) stable disease. Taking into account immune-related adverse events (irAEs), five (71.4%) of seven patients with thymoma and four (15.4%) of 26 patients with TC reported grade ≥ 3 irAEs, including myocarditis (three; 9.1%) [111].

As shown by these trials, due to the physiological function of the thymus and the fact that TETs, mainly thymomas, are associated with defective immune tolerance, the use of immunotherapy could expose TETs patients to an increased risk for developing irAEs compared with patients with other malignancies. The incidence of irAEs is high in thymoma patients but also those with TC detain a greater risk of developing severe irAEs. In fact, about 15% of patients, compared to 6% of patients affected by other cancers, develop grade ≥ 3 irAE, including potentially fatal myocarditis [111]. For this reason, an immune baseline check-up and a close monitoring of autoimmunity should be performed in TETs patients treated with ICIs, and their inclusion in clinical trials should be preferred. The detection of high tumor cells PD-L1 expression represents a strong rationale for using ICIs for treatment of TETs [114]. On the other hand, TMB, an emerging potential biomarker for ICIs efficacy, is very low in TETs [115, 116]. In a wide cohort of 100 thymomas and 69 TCs tissue expression of PD-L1, IDO and FOXP3 was analyzed and higher PD-L1 staining was detected in 36% of cases of both thymomas and TCs [114]. A work by Padda et al. [117], showed that TETs with higher PD-L1 expression detained a more aggressive histology (B3 thymomas and TCs) and worse prognosis. A meta-analysis about PD-L1 expression levels in TETs according to histological grade confirmed a significant higher PD-L1 positive rate in type B2/B3 thymoma and TC compared to the type A/AB/B1 thymoma group, suggesting that ICIs might be more effective for the former [118]. Interestingly, in a cohort of 43 patients affected by TCs, the PD-L1 tumor tissue staining increased after induction chemotherapy treatment [119]. This fact emphasizes that chemotherapy, but also targeted and epigenetic therapies [102, 120], holds immunomodulatory activities into the tumor microenvironment and that drug combinations with ICIs could be effective in a specific group of TETs patients. For example, belinostat, a pan-histone deacetylase inhibitor, detained immunomodulatory activity, leading to reduction in blood circulating Tregs and exhausted CD8^+^ T-cell population of TETs patients included in the phase II trial [120], suggesting a potential synergy between epigenetic drugs and immunotherapy.

Considering the overexpression of VEGFA and VEGFR-1 and -2 [121] and the frequent PD-L1 expression both in TETs [122], anti-VEGF agents and ICIs represent an alternative combination strategy.

In a cohort of TETs patients treated with sunitinib (a multiple receptor TKI active against VEGFR-1, -2 and -3, PDGFR-α, PDGFR-β and fibroblast growth factor receptor 1), higher expression of ICIs (i.e. CTLA4 and PD-1) was seen in circulating lymphocytes after sunitinib administration [102], suggesting its immuno-modulatory effect and a potential synergism with ICIs. That represented the rationale for phase II trials that are currently evaluating pembrolizumab plus sunitinib and axitinib plus avelumab, respectively, for treatment of patients with TC. Another phase I/II trial is ongoing to assess the safety, tolerability and efficacy of nivolumab and vorolanib in combination in patients with thoracic tumors including TC. Vorolanib is a new selective VEGFR/PDGFR TKI designed to minimize side effects compared to other anti-angiogenic multikinase inhibitors [123, 124].

Finally, a phase II trial is available for TC patients to evaluate the efficacy of epacadost and pembrolizumab. Epacadostat is a potent indoleamine 2,3-dioxygenase 1 (IDO1) enzyme inhibitor [125]. IDO1 plays a central role in immune regulation through the catabolism of tryptophan to kynurenine [126]. Kynurenine and others tryptophan metabolites induce suppression of effector CD8^+^ T cells function, increase activity of Tregs, inhibit NK-cells and promote expansion of DCs and MDSCs. Therefore, cancers that express high levels of IDO1 may elude immunosurveillance. In TETs, high expression of PD-L1 and IDO was observed in higher-grade forms of tumor histology [114] and for these reasons is reasonable to hypothesize a synergistic effect between epadacostat and pembrolizumab in patients affected by TC.

In conclusion, immunotherapy seems to be a promising therapeutic weapon in TETs, especially for TC. It is important to bear in mind that targeted therapies [127, 128] can also induce severe adverse effects and, due to their immune-modulation activity, potentially exacerbate irAEs linked to ICIs.

In order to better monitor TETs patients receiving immunotherapy, it would be appropriate to include patients in clinical trials and develop an appropriate and accurate monitoring plan that would allow early recognition of irAEs.

Discovering predictive factors, able to discriminate at baseline, before therapy start, patients more likely to develop potentially severe irAEs, would ultimately guide clinicians to the best therapeutic choice.

## Conclusions

Since the introduction of PD-L1 and PD-1 inhibitors into the field of NSCLC, several clinical trials attempted to readapt these drugs in other thoracic malignancies. Although a modest single-agent activity has been seen in SCLC, MPM, and TETs, the benefits of ICI monotherapy have remained below expectations. The subtle but substantial differences between these tumor types and within the same tumor type are most probably responsible for delaying the strong affirmation of ICI monotherapy in this setting. Last evidences showed that the molecular profile and the prognosis of these rarer thoracic malignancies might be better explained by a continuous model rather than by a canonical categorical subdivision [129, 130]. Similar to NSCLC, in which most of oncogene-driven tumors are highly resistant to ICIs, it is then improbable that all patients with SCLC, MPM, TETs will benefit from the same ICI strategy. In addition, it is now clear that the interaction between the tumor and the immune counterparts is considerably more complex and cannot be explained by just one immune checkpoint receptor. Spatial [131, 132], temporal, and therapeutic [133] factors can affect checkpoints (both PD-1, PD-L1 and PD-L2) expression in tumor microenvironment. Taking into account these factors in the context of robust international trials supported by a proper outcome selection and strong translational analyses will be mandatory in the next future. As first approvals of anti-PD-1/PD-L1 in SCLC and MPM and also results from early-phase trials have recently shown, combining ICIs with chemotherapy, different ICIs (such as those targeting CTLA4) or even with targeted therapies, might represent a valid solution to overcome resistance and to broaden the population of thoracic cancer patients who may benefit from immunotherapy. However, further insight into potential biomarkers (both at tumor and at patient level) [12, 130–133] as well as into the ideal timing/setting for immunotherapy is needed to get an upfront identification of patients who are likely to respond to these combination strategies and to avoid potentially harmful treatments. The reported toxicity profile of drug combinations draws clinical attention to both ongoing trials and real-world practice, with preexisting paraneoplastic disorders precluding the administration of ICIs combinations in some of these patients. Nevertheless, exploiting all advantages of ICIs in SCLC, MPM, and TETs is crucial, as very few other therapeutic options proved clinically relevant in these diseases.

## Abbreviations

AIRE: autoimmune regulator
CIK: cytokine-induced killer
CTLA4: cytotoxic T-lymphocyte-associated protein 4
DC: dendritic cell
DCR: disease control rate
DDR: DNA damage response
ES-SCLC: extensive-small cell lung cancer
FEZF2: FEZ family zinc finger 2
ICD: immunogenic cell death
ICIs: immune checkpoint inhibitors
IDO1: indoleamine 2,3-dioxygenase 1
irAEs: immune-related adverse events
ITT: intention-to-treat
MDSC: myeloid-derived suppressor cell
mOS: median overall survival
mPFS: median progression free survival
MPM: malignant pleural mesothelioma
NK-cell: natural killer cell
NSCLC: non-small cell lung cancer
ORR: objective response rate
OS: overall survival
PARP: poly (ADP-ribose) polymerase
PD-1: programmed cell death protein 1
PD-L1: programmed death-ligand 1
PD-L2: programmed death-ligand 2
PFS: progression-free survival
SCLC: small cell lung cancer
TC: thymic carcinomas
TETs: thymic epithelial tumors
TMB: tumor mutation burden
Tregs: regulatory T cells
VEGF: vascular endothelial growth factor

## Declarations

### Copyright

© The Author(s) 2021.
